# Assistive devices identified as most useful for people with Parkinson's disease – a patient-centered survey

**DOI:** 10.3389/fresc.2026.1898569

**Published:** 2026-09-25

**Authors:** Hollie Adams, Charles Babbush, Camilla Kilbane

**Affiliations:** 1 Case Western Reserve University School of Medicine, Cleveland, OH, United States; 2 Papa’s Path, Beachwood, OH, United States; 3 University Hospitals Cleveland Medical Center, Cleveland, OH, United States

**Keywords:** activities of daily living (ADL), assistive devices, assistive tools, Parkinson's disease, quality of life

## Abstract

**Objective:**

This study aimed to identify everyday assistive devices perceived as most useful by people with Parkinson's disease (PwP) and their caregivers.

**Methods:**

A 45-item web-based survey was distributed through the Parkinson & Movement Disorder Alliance website and an online educational event. The survey included four respondent-characteristic items, ratings of 40 assistive devices using a 5-point Likert scale ranging from “not useful” to “very useful,” and one free-response item.

**Results:**

A total of 293 online surveys were completed. The five devices with the highest percentages of “very useful” ratings were grab bars for shower (80%), grab bars (71%), electric toothbrushes (63%), jar openers (54%), and grabber tools (43%).

**Conclusions:**

Numerous assistive devices are available to make activities of daily living easier for PwP. Identifying which devices PwP and caregivers perceive as most useful may help clinicians prioritize practical recommendations and support greater independence in daily activities.

## 1 Introduction

Parkinson's disease (PD) can affect independence across many activities of daily living (ADLs), and a wide range of assistive technologies and devices are available to support everyday functioning and quality of life (QoL). Existing research on assistive technology for PwP has largely focused on walking and mobility, including gait monitoring (1–5), and tremor reduction (6–10), with more recent attention to technology-based tools such as exoskeletons and voice-activated devices (11, 12). Comparatively less research has examined readily available aids for other ADLs, including bathing and grooming, dressing, meal preparation, and feeding. This gap is particularly important because impairment in ADLs worsens with disease progression (13). Bathing/showering, dressing, and grooming/personal hygiene were among the most commonly affected basic self-activities in PwP (14), highlighting the need to better understand which readily available devices may help support independence in these everyday tasks.

As the impact of PD on daily function increases, identifying practical ways to support independence requires understanding which interventions PwP themselves find most helpful. This aligns with a growing emphasis on person-centered Parkinson's care, which prioritizes the active involvement of PwP in decisions about their health and daily functioning (15). Despite this shift, relatively few studies have systematically examined PwP perspectives on the usefulness of assistive devices. This focus drove the development of the present survey in collaboration with Papa's Path, a local nonprofit founded by a PwP, with the mission of enhancing quality of life and improving outcomes for patients and families. As a medical professional living with Parkinson's disease, CB initially developed the survey based on his lived experience navigating daily challenges. He experienced frustrations with visits to the neurology team focusing mostly on pharmacological management, with limited discussions about day-to-day activities. The identified gap in knowledge drove the development of the survey, as research on this topic has been limited. One study conducted in Bangkok, Thailand administered a 50-item survey to 222 PwP assessing major concerns across disease stage and age at symptom onset (16). Assistive devices were a commonly reported need, and the investigators concluded that many respondents were likely unaware of specialized devices. Similarly, a mixed-methods study incorporating qualitative interviews explored challenges PwP experience when handling cutlery (17). Despite reporting significant difficulty, few participants were aware of or used assistive devices.

Prior studies suggest that PwP may have limited awareness of available assistive devices, highlighting a need for practical information about which devices may be most beneficial for everyday functioning. The present study sought to capture PwP and caregiver perspectives on the perceived usefulness of assistive devices for everyday activities. Identifying the devices perceived as most useful may help inform clinical recommendations and support dissemination of practical information about available aids to PwP and their caregivers.

## 2 Methods

A comprehensive web-based anonymous survey incorporating questions regarding the perceived usefulness of various assistive devices in people with Parkinson's disease was developed. CB, a PwP, initially started collecting useful tools for activities of daily living, testing them out in his own daily life. This prompted the first development of the survey. Further input was obtained from CK, a movement disorder specialist, prior to deployment of the final survey. The goal was to learn from other PwP and get their perspectives on what tools they also found helpful. The survey consisted of 45 items. Four items collected respondent characteristics, including gender, respondent type, years since Parkinson's disease diagnosis, and prior use of occupational therapy services. Respondents then rated 40 individual assistive devices using a 5-point Likert scale ranging from “not useful” to “very useful” based on their perceived utility in day-to-day activities (Figure 1). The devices were organized into five categories: grooming and dressing aids, toileting aids, eating aids, kitchen and household items, and miscellaneous household aids. Both people with Parkinson's disease and their caregivers could complete the survey, as the perspective of a PwP or caregiver may differ. A final free response item invited respondents to identify additional useful devices not included in the survey. The survey was disseminated in collaboration with the Parkinson & Movement Disorder Alliance (PMD Alliance), which made the survey available through its website, via emails and during an online educational event with CB and CK. Responses were obtained predominantly from people in the United States. The top five assistive devices were determined by the highest percentages of “very useful” ratings and the highest combined percentages of “very useful + somewhat useful” ratings (Table 1).

**Figure 1:**
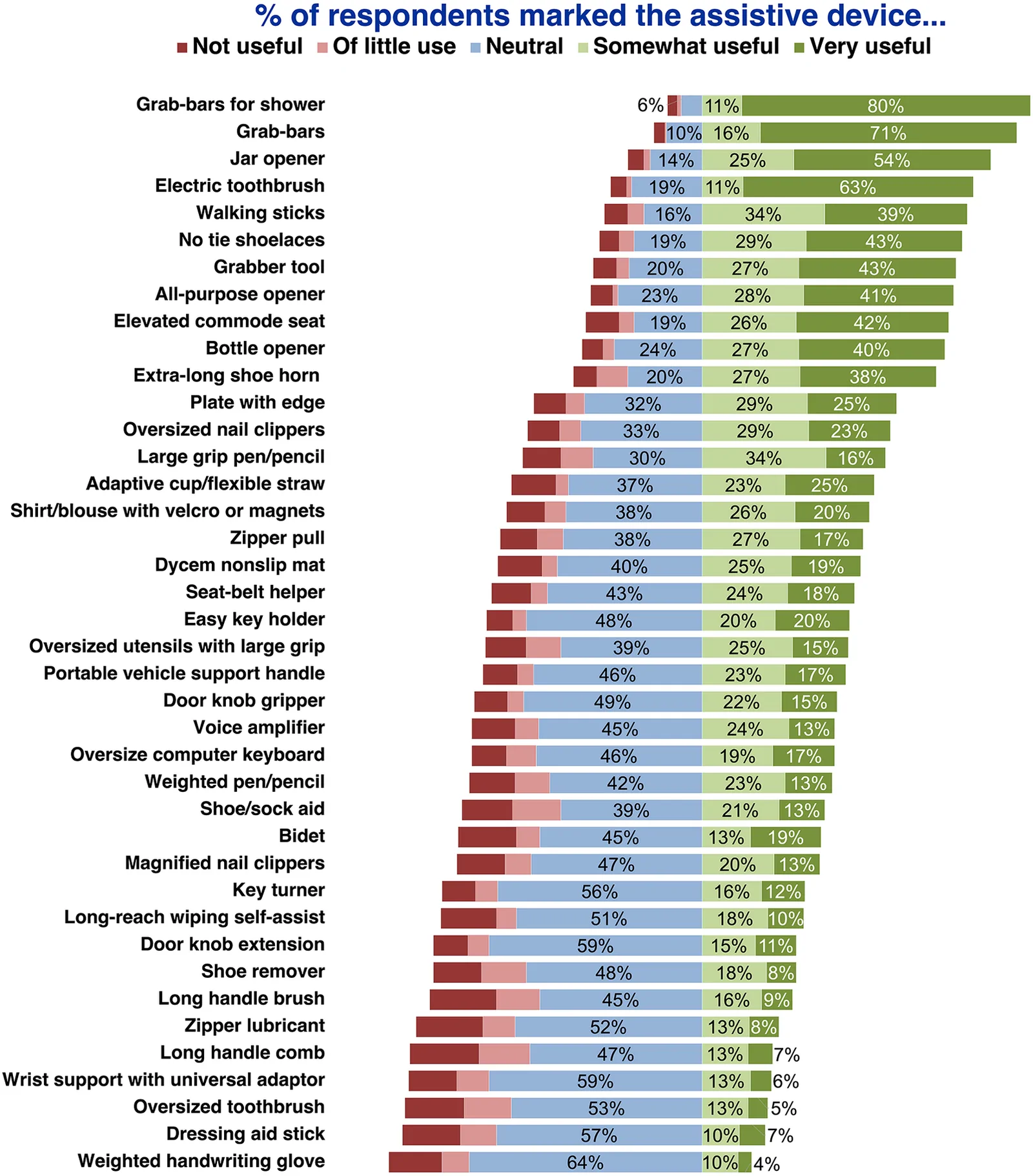
Distribution of perceived usefulness of 40 assistive devices among respondents with Parkinson's disease and their caregivers. Devices were rated using a 5-point Likert scale ranging from “not useful” to “very useful” based on their utility in day-to-day activities.

**Table 1:** Top five aids.

Highest Percentages of “Very Useful”	
Grab bars for shower	80%
Grab bars	71%
Electric toothbrush	63%
Jar opener	54%
Grabber tool	43%
Highest Combined Percentages of “Very Useful + Somewhat Useful”	
Grab bars for shower	90%
Grab bars	87%
Jar opener	79%
Electric toothbrush	75%
Walking sticks	73%

## 3 Results

Of the 293 respondents, 216 identified as PwP, including 214 who directly selected this option and two respondents who selected “other” and clarified their status as PwP in the write-in response. Caregivers formed the second-largest group, with 73 respondents, including 72 who directly identified as caregivers and one former caregiver whose partner had passed away. The remaining four respondents included an occupational therapist, a certified personal trainer, a friend of a PwP, and a sister of a PwP. Across all respondents, 34% were male and 66% were female. Among respondents reporting years since Parkinson's diagnosis, 37% reported 0–5 years, 29% reported 6–10 years, and 33% reported more than 10 years since diagnosis. Most respondents (61%) had not worked with an occupational therapist (OT) to address independence in or energy conservation of ADLs, while 39% reported receiving OT services.

The five aids with the highest percentages of “very useful” ratings were grab bars for shower (80%), grab bars (71%), electric toothbrushes (63%), jar openers (54%), and grabber tools (43%). When “very useful” and “somewhat useful” responses were combined, the five highest-rated aids were grab bars for shower (90%), grab bars (87%), jar openers (79%), electric toothbrushes (75%), and walking sticks (73%) (Table 1). Thus, walking sticks replaced grabber tools among the top five devices when “somewhat useful” responses were included, and the relative ordering of several devices also changed.

Subgroup analyses for the top five aids were conducted across various demographics, including female vs. male respondents, years since diagnosis (0–5 years, 6–10 years, vs. > 10 years), OT users vs. non-OT users, and PwP vs. caregivers. Overall, very little variation was observed in the top five aids across subgroup analyses. Across all groups, for both “very useful” and “very useful + somewhat useful”, grab bars for shower, grab bars, electric toothbrushes, and jar openers were consistently represented in the top five aids. Minor differences were observed when comparing caregivers' and PwP responses. Caregivers rated the elevated commode as more helpful than PwP did; it ranked among the top five most useful aids for caregivers but not for PwP. When stratified by years since diagnosis, the top five aids rated as “very useful + somewhat useful” were identical in both the 0–5 year and 6–10-year groups: grab bars for shower, electric toothbrushes, grab bars, jar openers, and walking sticks. Among PwP with a diagnosis of more than 10 years, the top five aids were largely consistent; however, walking sticks no longer appeared in this group's top five most helpful devices.

## 4 Discussion

Parkinson's care has traditionally been fragmented, with poor interdisciplinary collaboration. In addition, it has typically been more “reactive”, focusing on solving problems when they arise. More recently a more “patient centric” and later “person centric” health care model has been proposed. In a person centric health care model, paternalistic care and advice from health care professionals should be replaced by a participatory health care model with a focus on self-management by well informed and empowered PwP (15). Part of this person centric care model should include education and research on how to improve the impact of PD on daily activities. Impairment in ADLs worsens with disease progression (13). Despite this push, there have been very few studies asking PwP what assistive devices actually help them in their day-to-day functioning. The largest focus has been on assistive devices for gait function (1–5), yet many simple and effective aids exist for other everyday tasks. Despite this, knowledge about these products may be limited. More studies should be conducted, asking PwP for their perspective and their lived experiences. The most reported basic self-care activities affected by PD symptoms were bathing/showering (69% of participants), dressing (66%), and grooming/personal hygiene (59%) in a recently developed instrument to assess daily living function in PD (14). Given the frequency of these symptoms, clinicians should be more readily aware of tools which make these ADLs less challenging. Oftentimes, PwP will be referred to allied health professionals such as occupational therapists, physical therapists, and speech-language pathologists who can evaluate needs and offer tailored recommendations, including assistive devices. Among our respondent population, a large portion (61%) had not seen an occupational therapist to address independence in or energy conservation during ADLs; therefore, care providers for PwP should be more formally educated and aware of these different types of devices. Proactively educating patients about these devices can improve self-agency and potentially reduce caregiver burden by allowing the PwP greater independence. Future directions include analyzing the cost-effectiveness of these devices and improving their coverage by health insurance. We also chose to include caregivers' input in our survey. Caregivers of PwP often help with a range of tasks, including ADLs, and their input on this topic is therefore highly valuable. PD has long been associated with high rates of caregiver burden, which has been linked to adverse health outcomes for both PwP and caregivers (18). We also suspected that by allowing caregivers to respond to the survey, we could capture what aids were deemed more helpful in more advanced PD, when assistance with more ADLs is needed.

This study has several limitations that should be considered when interpreting the results. The survey did not include images or detailed descriptions of the assistive devices. This absence of comprehensive information may have affected respondents' ability to accurately assess the usefulness of each aid, especially those that are not widely known. We asked participants to assess the usefulness of aids even in the absence of prior use. These hypothetical evaluations could potentially lead to less accurate or reliable responses. The survey did not include a mechanism for respondents to indicate prior experience with these devices. This could have led to inconsistent rating approaches, as respondents may have based their assessments on actual use, while others relied on speculation. To address these limitations, future surveys could include detailed descriptions and images of each assistive device to ensure participants have a clear understanding of the aids they are evaluating. Incorporating questions about respondents' familiarity with and prior use of each device can better contextualize their rating. Furthermore, a more accurate assessment of a device's true usefulness would involve having PwP use the device for a set period and then rate its effectiveness. However, implementing such a trial would introduce logistical challenges and potentially limit participant reach. The survey was also developed based on a single PwP experience, with subsequent input from a movement disorder neurologist. Feedback from more PwP could have been obtained prior to distribution of the survey, to gain a wider PwP perspective. Despite these limitations, we feel that the results of this survey are important. Many of the tools identified as useful are low cost and can easily be deployed across wide socioeconomic backgrounds. Over 60% of the respondents had not seen an occupational therapist, highlighting the need for the neurology care providers to include tool recommendations in their practices. By integrating PwP perspectives, healthcare providers can offer more informed recommendations, centering PwP decision-making abilities about which aids are most beneficial for their individual needs.

## Data Availability

The raw data supporting the conclusions of this article will be made available by the authors, without undue reservation.
